# Formation of Chlorotriophenoxy Radicals from Complete Series Reactions of Chlorotriophenols with H and OH Radicals

**DOI:** 10.3390/ijms160818714

**Published:** 2015-08-11

**Authors:** Fei Xu, Xiangli Shi, Qingzhu Zhang, Wenxing Wang

**Affiliations:** Environment Research Institute, Shandong University, Jinan 250100, China; E-Mails: xufei@sdu.edu.cn (F.X.); 123songchuan@163.com (X.S); wxwang@sdu.edu.cn (W.W.)

**Keywords:** chlorothiophenols, chlorothiophenoxy radicals, H radicals, OH radicals, reaction mechanism, rate constants

## Abstract

The chlorothiophenoxy radicals (CTPRs) are key intermediate species in the formation of polychlorinated dibenzothiophenes/thianthrenes (PCDT/TAs). In this work, the formation of CTPRs from the complete series reactions of 19 chlorothiophenol (CTP) congeners with H and OH radicals were investigated theoretically by using the density functional theory (DFT) method. The profiles of the potential energy surface were constructed at the MPWB1K/6-311+G(3df,2p)//MPWB1K/6-31+G(d,p) level. The rate constants were evaluated by the canonical variational transition-state (CVT) theory with the small curvature tunneling (SCT) contribution at 600–1200 K. The present study indicates that the structural parameters, thermal data, and rate constants as well as the formation potential of CTPRs from CTPs are strongly dominated by the chlorine substitution at the *ortho*-position of CTPs. Comparison with the study of formation of chlorophenoxy radicals (CPRs) from chlorophenols (CPs) clearly shows that the thiophenoxyl-hydrogen abstraction from CTPs by H is more efficient than the phenoxyl-hydrogen abstraction from CPs by H, whereas the thiophenoxyl-hydrogen abstraction from CTPs by OH is less impactful than the phenoxyl-hydrogen abstraction from CPs by OH. Reactions of CTPs with H can occur more readily than that of CTPs with OH, which is opposite to the reactivity comparison of CPs with H and OH.

## 1. Introduction

Polychlorinated dibenzothiophenes (PCDTs) and polychlorinated thianthrenes (PCTAs) are analogues of dibenzofurans (PCDFs) and polychlorinated dibenzo-*p*-dioxins (PCDDs), respectively, in which the oxygen atoms are substituted by the sulfur atoms. Therefore, they have similar geochemical behavior, toxicity, and physicochemical properties in the environment [1,2,3,4,5,6,7,8]. PCDT/TAs have been detected in various environmental samples such as stack gases, incineration of municipal waste, pulp mill effluents, soil and sediments, petroleum refineries, petroleum spills, pine needles, and some aquatic organisms [1,5,9,10,11,12]. The long-term adverse environmental effects of PCDT/TAs have been at the forefront of public and regulatory concern, and information about the formation mechanisms of PCDT/TAs in the environment is required.

PCDT/TAs were never intentionally synthesized for commercial purposes, but are formed as byproducts from the chemical processes that are similar to those resulting in the formation of PCDD/Fs. The major known sources of PCDT/TAs in environment are combustion, emissions from municipal and hazardous waste incinerators as well as industrial incinerators [13,14]. High correlation between concentrations of PCDT/TAs and PCDD/Fs in the environmental samples revealed their similar formation mechanism under the pyrolysis or combustion conditions [11,15,16]. The most direct route to the formation of PCDT/TAs is the gas-phase reaction of chemical precursors.

Chlorophenols (CPs) are structurally similar to PCDD/Fs and the most direct precursors of PCDD/Fs [17,18,19,20,21,22]. Similarly, chlorothiphenols (CTPs) are structurally similar to PCDT/TAs and have been demonstrated to be the predominant precursors or key intermediates of PCDT/TA formation [23,24,25,26]. For instance, pentachlorothiophenol, an important additive in the vulcanization process of rubber in the tire industry, represents an important precursor for the formation of octachlorodibenzothiophene (octaCDT), heptachlorothianthrene (heptaCTA), and octachlorothianthrene (octaCTA) [26]. CTPs have been widely used in large quantities in various chemical industries, such as in manufacturing of dyes, insecticides, printing inks, pharmaceuticals, and polyvinyl chloride [27]. CTPs are toxic and hazardous to human health and environment due to the presence of sulfur and chlorine [28,29]. Variously halogenated derivatives of phenol and thiophenol were subjected to analysis of their inhibitory effect on human cytochrome P450 (CYP) 2E1, which showed that dichlorothiophenols have stronger potent inhibitory activities than dichlorophenols, and the toxicity of CTPs are influenced by chlorine substitution pattern [29].

Similar to the formation of PCDD/Fs from CP precursors, the gas-phase formation of PCDT/TAs from CTP precursors was also proposed involving radical-radical coupling of two CTPRs and radical-molecule recombination of CTPR and CTP. The recent works have shown that radical-radical coupling are more competitive thermodynamically than radical-molecule recombination for the PCDT/TA formation [24,25,30,31,32,33]. The dimerization of CTPRs is the major PCDT/TA formation pathway [24,25]. Thus, the formation of CTPRs is the initial and key step involved in the formation of PCDT/TAs. Under the pyrolysis or combustion conditions, CTPRs can be formed through loss of the triophenoxyl-hydrogen via unimolecular, bimolecular, or possibly other low-energy pathways (including heterogenous reactions). The unimolecular reaction includes the decomposition of CTPs with the cleavage of the S–H bond. The bimolecular reactions include attack by H, OH, O(^3^P), or Cl under high-temperature oxidative conditions. As yet, very little work has been done at the high temperatures relevant to these reactions.

In recent research from this laboratory, we investigated the formation of chlorophenoxy radicals (CPRs) from the reactions of CPs with H and OH radicals [34,35], based on the kinetic model conclusion that PCDD/F yields are most sensitive to the reactions of CPs with H and OH [36]. Thus, as part of our ongoing work in the field, the thiophenoxyl-hydrogen abstraction from CTPs with H and OH are naturally expected to play the most central role in the formation of CTPRs. Here, therefore, we performed a direct density functional theory (DFT) kinetic study on the formation of CTPRs from the complete series reactions of 19 CTP congeners with H and OH radicals. We also studied the reactions of thiophenol with H and OH radicals for comparison. The effect of the chlorine substitution pattern on the structures, energies, and rate constants is discussed. The formation potential of CTPRs from CTPs with H and OH are compared with that of CPRs from CPs with H and OH, respectively.

## 2. Results and Discussion

**Figure 1:**
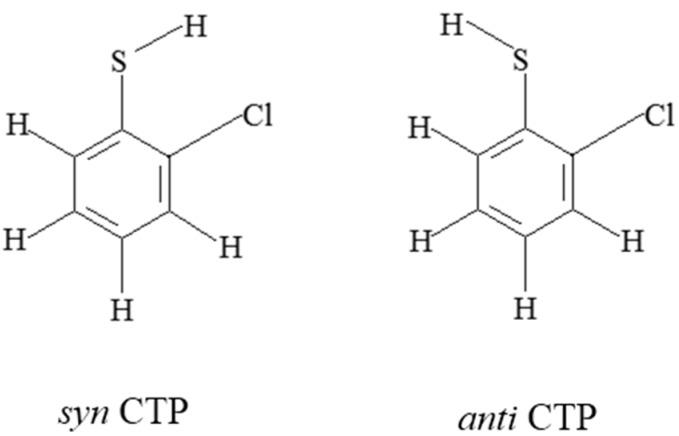
syn and anti conformers of CTP.

Due to the different substitution pattern of thiophenol, chlorothiophenols have 19 congeners, including three monochlorothiophenols (2-CTP, 3-CTP and 4-CTP), six dichlorothiophenols (2,3-DCTP, 2,4-DCTP, 2,5-DCTP, 2,6-DCTP, 3,4-DCTP and 3,5-DCTP), six trichlorothiophenols (2,3,4-TCTP, 2,3,5-TCTP, 2,3,6-TCTP, 2,4,5-TCTP, 2,4,6-TCTP and 3,4,5-TCTP), three tetrachlorothiophenols (2,3,4,5-TeCTP, 2,3,4,6-TeCTP and 2,3,5,6-TeCTP), and pentachlorothiophenols (PCTP). Due to the asymmetric chlorine substitution, there are *syn* and *anti*-conformers for 2-CTP, 3-CTP, 2,3-DCTP, 2,4-DCTP, 2,5-DCTP, 3,4-DCTP, 2,3,4-TCTP, 2,3,5-TCTP, 2,3,6-TCTP, 2,4,5-TCTP, 2,3,4,5-TeCTP and 2,3,4,6-TeCTP, respectively. The conformer with the sulfydryl-hydrogen facing the closest neighboring Cl is labeled as the *syn*-conformer and otherwise the *anti*-conformer (Figure 1). For a given CTP, the *syn*-conformer is about 0.5 kcal/mol more stable than the corresponding *anti* form, suggesting a stabilization effect because of intramolecular hydrogen bonding. So, throughout this paper, CTPs denote the *syn*-conformers. 

The structures of CTPs along with the structure of thiophenol are presented in the Figure S1 of Supplementary Materials. The structural parameters of CTPs are strongly influenced by the *ortho*-substituted chlorine regardless of the number of chlorine substituents. There exists weak intramolecular hydrogen bonding in the *ortho*-substituted CTPs. The lengths of the intramolecular hydrogen bonds are from 2.391 to 2.490 Å. No such intramolecular hydrogen bonding forms in the *anti*-conformers except those with chlorine substitutions at both *ortho*-positions. The C–S bonds in CTPs are from 1.749 to 1.761 Å, which are longer than the C–S double bond and shorter than the C–S single bond. The C–S bonds lengths (1.749–1.754 Å) in the *ortho*-substituted CTPs are consistently shorter than those for all *nonortho* forms (1.756–1.761 Å). The structures of CTPRs along with the structure of thiophenoxy radical are shown in the Figure S2 of Supplementary Materials.

Comparison of C–S bond lengths presented in Figure S1 with the C–O bond lengths of CPs in our previous study [34] clearly shows that C–S bond lengths in CTPs are longer than the C–O bond length of CPs (1.331–1.352 Å). Similarly, S–H bond lengths in CTPs (1.332 or 1.333 Å) are longer than the O–H bond length of CPs (0.955–0.960 Å).  Table S1 shows the NBO charge of S and H of CTPs (NBO_S_ and NBO_H_), NBO charge of O and H of CPs (NBO_O_ and NBO_H_) and HOMO-LOMO gap of CTPs and CPs at MPWB1K/6-31+G(d,p) level. At a given CP and CTP, the NBO_O_ of CP is more negative than the NBO_S_ of CTP, and the NBO_H_ of CP is more positive than that of CTP. This means the O atom in CP have stronger nucleophilicity than the S atom in CTP, *i.e*., the O–H bond strength in CP is stronger than the S–H bond in CTP. In addition, the HOMO-LOMO gap of CP is larger than that of CTP, which reconfirms that CP is more stable than CTP. Figure S3 depicts the electron density of 2-CTP/2-CP and 3-CTP/3-CP at MPWB1K/6-311+G(3df,2p) level. The S–H and C–S bond lengths of thiophenol were also studied by Larsen *et al.* using both experimental investigation and *ab initio* molecular calculations at B3LYP/aug-cc-pVQZ, MP2(full)/aug-cc-pVTZ and MP2(full)/aug-cc-pVQZ levels [37]. The S–H bond length of 1.333 Å and C–S bond length of 1.761 Å obtained in our study at the MPWB1K/6-31+G(d,p) level are in good agreement with the experimental value of 1.333 Å and 1.773 Å with the discrepancy less than 1.0% [37]. Compared with Larsen’s calculation values of S–H and C–S bond lengths, our MPWB1K/6-31+G(d,p) results are slightly closer to the values at MP2(full)/aug-cc-pVTZ (1.334 Å for S–H bond and 1.763 Å for C–S bond) and MP2(full)/aug-cc-pVQZ levels (1.332 Å for S–H bond and 1.760 Å for C–S bond) than those at B3LYP and aug-cc-pVQZ levels (1.341 Å for S–H bond and 1.779 Å for C–S bond) [37]. 

### 2.1. Reactions of CTPs with H 

**Figure 2:**
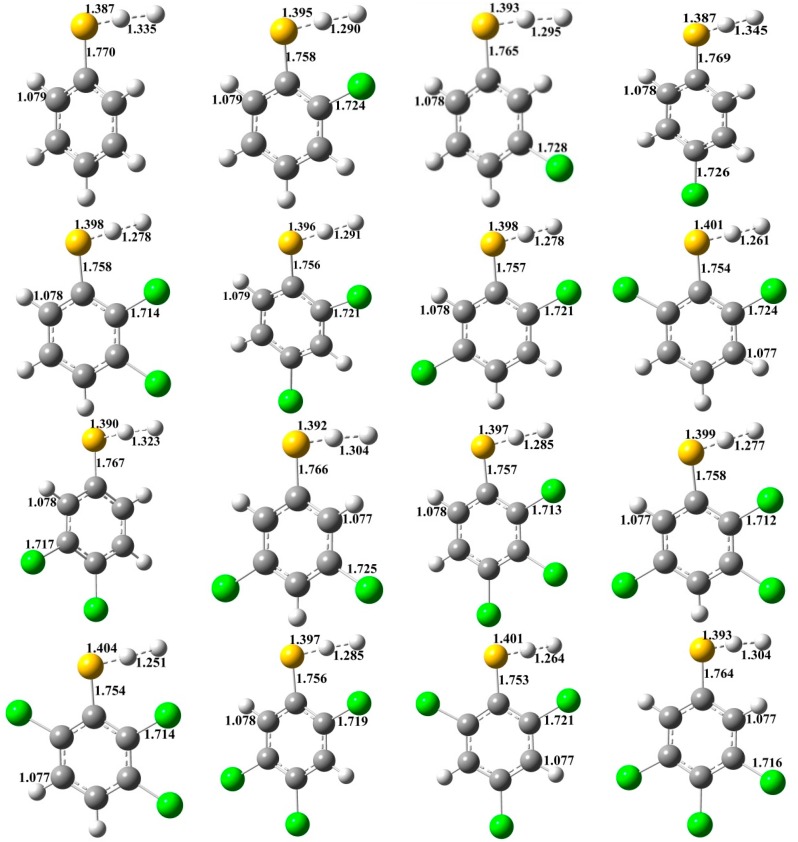

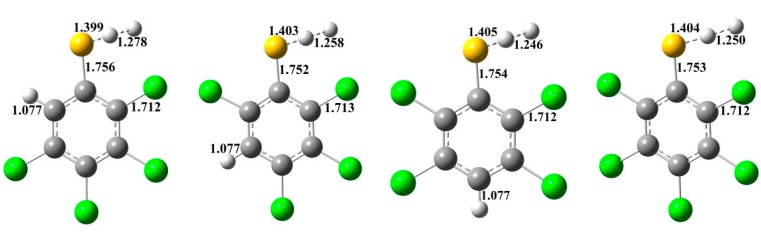
MPWB1K/6-31+G(d,p) optimized geometries for the transition states of the thiophenoxyl-hydrogen abstraction from CTPs by H. Distances are in angstroms. Gray sphere, C; White sphere, H; Yellow sphere, S; Green sphere, Cl. (For interpretation of the references to color in this figure legend, the reader is referred to the web version of this article.

The formation of CTPRs from the reactions of CTPs *with* H proceeds via a direct hydrogen abstraction mechanism. The structures of the transition states were located at the MPWB1K/6-31+G(d,p) level and shown in Figure 2. The H–H and C–S bonds in CTPs with *ortho*-substitution (1.246–1.291 Å for H–H, and 1.752–1.758 Å for C–S) are systematically shorter than those without *ortho*-substitution (1.295–1.345 Å for H–H, and 1.764–1.770 Å for C–S), respectively. Besides, all the *ortho*-substituted transition states have relative longer S–H bonds lengths (1.395–1.405 Å) compared to those without *ortho*-substitution (1.387–1.393 Å). Table 1 gives the potential barriers and reaction heats obtained at the MPWB1K/6-311+G(3df,2p)//MPWB1K/6-31+G(d,p) level. The formation of CTPRs from the reactions of CTPs with H is strongly exothermic. Table 1 shows that the potential barriers are significantly correlated with the position of the chlorine substitution at the thiophenolicring, but not with the number of chlorine substituents. For example, for dichlorothiophenols, the potential barriers of the thiophenoxyl-hydrogen abstraction from 2,3-DCTP, 2,4-DCTP, 2,5-DCTP and 2,6-DCTP are higher than those from 3,4-DCTP and 3,5-DCTP. For trichlorothiophenols, the potential barriers of the phenoxyl-hydrogen abstraction from 2,3,4-TCTP, 2,3,5-TCTP, 2,3,6-TCTP, 2,4,5-TCTP and 2,4,6-TCTP are higher than that from 3,4,5-TCTP. Obviously, for a given number of chlorine substitutions, the potential barriers for the thiophenoxyl-hydrogen abstraction from the *ortho*-substituted CTPs are consistently higher than those for other structural conformers. The chlorine substitution at the *ortho*-position can lower the barrier heights of thiophenoxyl-hydrogen abstraction from CTPs by H. Intramolecular hydrogen bonding appears to stabilize the CTPs and reduce the reactivity of S–H bonds in CTPs with the *ortho*-substitution. A similar result was also observed in our previous study of CPs with H [34].

**Table 1:** Potential barriers ∆*E* (in kcal/mol), reaction heats ∆*H* (in kcal/mol, 0 K), imaginary frequencies (in cm^−1^) of the transition states, and the S–H bond dissociation energies *D*_0_ (S–H) (in kcal/mol) for the thiophenoxyl-hydrogen abstraction from CTPs by H.

CTP	∆*E*	∆*H*	ν	*D*_0_ (S-H)
Thiophenol	2.50	−14.16	−789i	86.51
2-CTP	3.42	−14.43	−879i	86.24
3-CTP	2.76	−13.94	−871i	86.73
4-CTP	2.31	−23.50	−775i	77.16
2,3-DCTP	3.56	−14.71	−922i	85.59
2,4-DCTP	3.44	−21.52	−889i	79.15
2,5-DCTP	3.64	−12.90	−918i	87.76
2,6-DCTP	4.38	−15.68	−966i	84.99
3,4-DCTP	2.65	−22.68	−820i	77.98
3,5-DCTP	3.00	−13.74	−851i	86.92
2,3,4-TCTP	3.21	−21.10	−903i	79.57
2,3,5-TCTP	3.70	−14.65	−917i	86.01
2,3,6-TCTP	4.43	−13.03	−994i	87.64
2,4,5-TCTP	3.48	−20.94	−897i	79.72
2,4,6-TCTP	4.27	−20.12	−965i	80.55
3,4,5-TCTP	2.67	−22.12	−860i	78.55
2,3,4,5-TeCTP	3.17	−20.57	−921i	80.10
2,3,4,6-TeCTP	4.37	−19.65	−980i	81.02
2,3,5,6-TeCTP	4.52	−16.48	−1010i	84.18
PCTP	4.62	−18.98	−1000i	81.69

In order to further investigate the relative strength of the S–H bonds in CTPs, we also calculated the S–H bond dissociation energies *D*_0_ (S–H). The values of *D*_0_ (S–H) obtained at the MPWB1K/6-311+G(3df,2p)//MPWB1K/6-31+G(d,p) level are summarized in Table 1. *D*_0_ (S–H) of 2-CTP is higher than those of 4-CTP. Similarly, *D*_0_ (S–H) of 2,3-DCTP, 2,4-DCTP, 2,5-DCTP and 2,6-DCTP are higher than that of 3,4-DCTP. *D*_0_ (S–H) of 2,3,4-TCTP, 2,3,5-TCTP, 2,3,6-TCTP, 2,4,5-TCTP and 2,4,6-TCTP are higher than that of 3,4,5-TCTP. The chlorine substitution at the *ortho*-position appears to increase the strength of the S–H bonds in CTPs. However, for a given number of chlorine substitutions, the S–H bond dissociation energies in CTPs with *ortho*-substitution are not consistently larger than those without *ortho-*substitution. For example, *D*_0_ (S–H) of 2-CTP is smaller than that of 3-CTP. *D*_0_ (S–H) of 2,3-DCTP, 2,4-DCTP and 2,6-DCTP are smaller than that of 3,5-DCTP. Chlorine in an aromatic ring is traditionally recognized as an electron-withdrawing group. The intramolecular hydrogen bonding in the *ortho*-substituted CTPs as well as the inductive effect of the electron-withdrawing chlorine and steric effect may ultimately be responsible for the relative strength of the S–H bonds in CTPs.

It is interesting to compare the thiophenoxyl-hydrogen abstraction from CTPs by H with the phenoxyl-hydrogen abstraction from CPs by H [34]. For a given CTP, the potential barrier for the thiophenoxyl-hydrogen abstraction from CTP by H is about 8–11 kcal/mol lower than phenoxyl-hydrogen abstraction from corresponding CP by H [34]. In addition, the thiophenoxyl-hydrogen abstraction by H is more exothermic than the phenoxyl-hydrogen abstraction by H [34]. This indicates that the thiophenoxyl-hydrogen abstraction from CTPs by H can occur more promptly than the phenoxyl-hydrogen abstraction CPs by H.

### 2.2. Reactions of CTPs with OH

**Figure 3:**
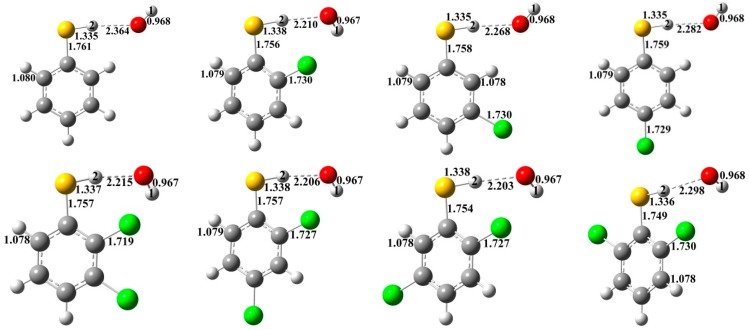

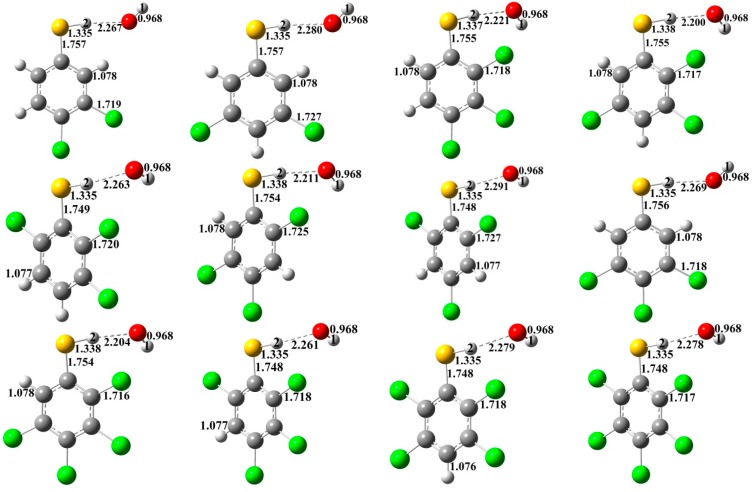
MPWB1K/6-31+G(d,p) optimized geometries for the prereactive intermediates of the thiophenoxyl-hydrogen abstraction from CTPs by OH. Distances are in angstroms. Gray sphere, C; White sphere, H; Yellow sphere, S; Red sphere, O; Green sphere, Cl. (For interpretation of the references to color in this figure legend, the reader is referred to the web version of this article.).

For thiophenoxyl-hydrogen abstraction from CTPs by OH radical, prereactive intermediates are formed before the transition state. The structures of the prereactive intermediates are presented in Figure 3. As shown in Figure 3, conformations of the intermediates are difference between *ortho*-substituted structures and *nonortho*-substituted structures. In the *ortho*-substituted intermediates, H(1) atom is at the *trans*-position of O with respect to the O–H(2) bond. In contrast, H(1) atom is at the *cis*-position of O in the intermediates without *ortho*-substitution. In addition, the *ortho*-substitution also has an effect on other structural parameters, such as the H(1)–O, H(2)–S and C–S bonds. For example, all the *ortho*-substituted intermediates have relatively shorter C–S bond distances (1.748–1.757 Å) compared to those without *ortho*-substitution (1.756–1.761Å). The relative energy, ∆*E*_IM_, of the intermediate with respect to the total energy of the corresponding CTP and OH is listed in Table 2.

**Table 2:** The relative energies of the intermediates ∆*E*_IM_ (in kcal/mol), potential barriers ∆*E*_TS_ (in kcal/mol), reaction heats ∆*H* (in kcal/mol, 0 K), imaginary frequencies (in cm^−1^) of the transition states for the triophenoxyl-hydrogen abstraction from CTPs by OH.

CTP	Δ*E*IM	Δ*E*TS	Δ*H*	ν
Thiophenol	−1.28	7.03	−27.69	−2237i
2-CTP	−0.90	8.67	−27.96	−2521i
3-CTP	−1.12	7.64	−27.47	−2484i
4-CTP	−1.38	6.99	−37.03	−2267i
2,3-DCTP	−0.59	9.29	−28.24	−2588i
2,4-DCTP	−0.73	8.80	−35.05	−2518i
2,5-DCTP	−0.77	9.20	−27.97	−2633i
2,6-DCTP	−0.99	10.27	−29.21	−2682i
3,4-DCTP	−1.42	7.39	−36.21	−2484i
3,5-DCTP	−1.48	8.13	−27.27	−2623i
2,3,4-TCTP	−0.91	9.10	−34.63	−2584i
2,3,5-TCTP	−0.64	9.48	−28.18	−2683i
2,3,6-TCTP	−0.85	10.48	−26.56	−2753i
2,4,5-TCTP	−0.78	8.98	−34.47	−2594i
2,4,6-TCTP	−1.00	9.95	−33.65	−2680i
3,4,5-TCTP	−1.65	7.66	−35.65	−2594i
2,3,4,5-TeCTP	−1.00	9.29	−34.10	−2680i
2,3,4,6-TeCTP	−1.04	10.18	−33.18	−2732i
2,3,5,6-TeCTP	−1.40	10.85	−30.01	−2792i
PCTP	−1.71	10.55	−32.51	−2796i

**Figure 4:**
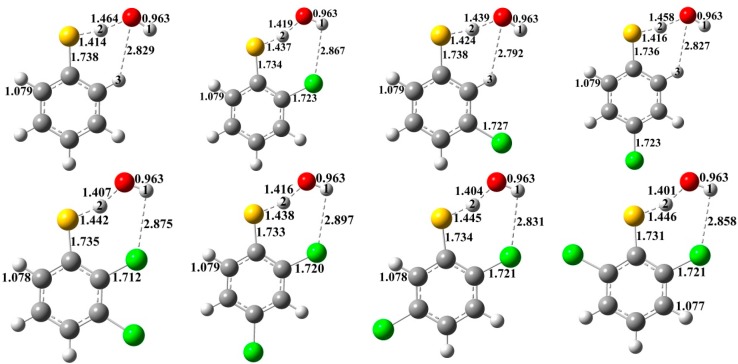

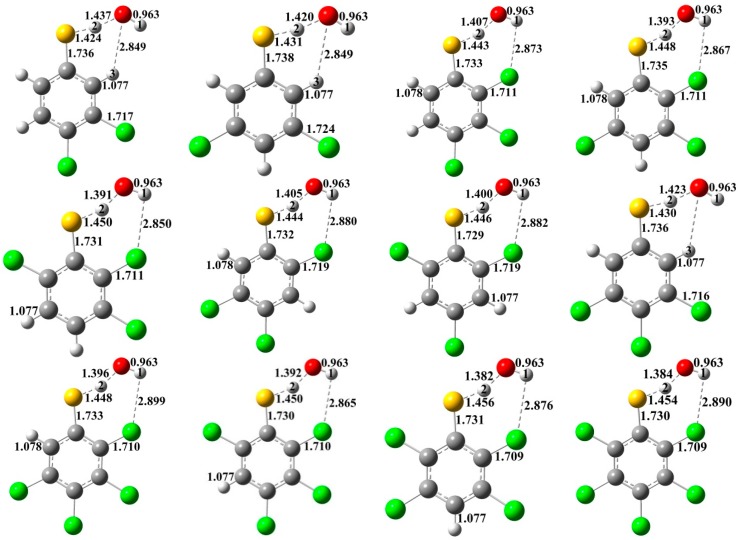
MPWB1K/6-31+G(d,p) optimized geometries for the transition states of the thiophenoxyl-hydrogen abstraction from CTPs by OH. Distances are in angstroms. Gray sphere, C; White sphere, H; Yellow sphere, S; Red sphere, O; Green sphere, Cl. (For interpretation of the references to color in this figure legend, the reader is referred to the web version of this article.).

The structures of the transition states are depicted in Figure 4. As shown in Figure 4, H(1) atom is at the *trans*-position of O with respect to the O–H(2) bond in the transition states with or without *ortho-*substitution. There are exit weak intramolecular hydrogen bondings in all the structures, which are governed by the chlorine substitution pattern. In the *ortho*-transition states, the intramolecular hydrogen bondings are between H(1) and *ortho* Cl atoms. In the transition states without *ortho*-substitution, the intramolecular hydrogen bondings are between O and H(3) atoms. The hydrogen bond can lower the energy of the transition state, *i.e*., lower the reaction potential barrier. Besides, the *ortho-*substitution also impacts other essential structural parameters of the transition states. Generally, the breaking S–H(2) bonds in the *ortho*-substituted transition states (1.437–1.456 Å) are longer than those without *ortho*-substitution (1.414–1.431 Å). The forming O–H(2) bonds in the transition states with *ortho*-substitution (1.382–1.419 Å) are shorter than those without *ortho*-substitution (1.420–1.464 Å).

The potential barriers and reaction heats calculated at the MPWB1K/6-311+G(3df,2p)//MPWB1K/6-31+G(d,p) level are shown in Table 2. In particular, the potential barrier is the relative energy of the transition state with respect to the total energy of the separated reactants (the corresponding CTP and OH), without considering the very shallow prereactive intermediate. It can be seen from Table 2 that the potential barriers for the thiophenoxyl-hydrogen abstraction from the *ortho*-substituted CTPs by OH radicals consistently are higher than those from CTPs without *ortho*-substitution. This reaffirms the conclusion above that the chlorine substitution at the *ortho* position increases the strength of the S–H bonds and decreases its reactivity.

It is also necessary to compare the thiophenoxyl-hydrogen abstraction from CTPs by OH with the phenoxyl-hydrogen abstraction from CPs by OH of our previous study [35]. For a given chlorotriophenol, the potential barrier for the thiophenoxyl-hydrogen abstraction from CTPs by OH is about 5–8 kcal/mol higher than phenoxyl-hydrogen abstraction from CPs by OH [35], which indicates that the thiophenoxyl-hydrogen abstraction from CTPs by OH are more difficult to happen than the phenoxyl-hydrogen abstraction CPs by OH. Compared to the stereo configurations of the transition states from CPs with OH, the transition states from CTPs with OH have the planar structure with all the S, O, H(1), and H(2) atoms almost in the same plane as the benzene ring. This can higher the energy of transition states, *i.e*., higher the potential energy of CTPs with OH radicals. 

Comparison of the values presented in Table 1 and Table 2 shows that for a given CTP, the potential barrier for the thiophenoxyl-hydrogen abstraction by OH is about 4–6 kcal/mol lower than that of the thiophenoxyl-hydrogen abstraction by H, which indicates that the thiophenoxyl-hydrogen abstraction from CTPs by OH is less efficient than the thiophenoxyl-hydrogen abstraction by H. This is completely on the contrary to the fact of phenoxyl-hydrogen abstraction CPs by OH is more impactfulthan the phenoxyl-hydrogen abstraction by H [35].

### 2.3. Rate Constant Calculations 

Canonical variational transition state theory (CVT) with small-curvature tunneling (SCT) contribution has been successfully performed for formation of CPRs from the complete series reactions of 19 CP congeners with H and OH radicals [34,35], and is an efficient method to calculate the rate constants. In this study, we used this method to calculate the rate constants for the formation of CTPRs from the complete series reactions of 19 CTP congeners with H and OH radicals over a wide temperature range of 600–1200 K, as shown in Tables S2 and S3 of Supplementary Materials, respectively. This temperature range covers the possible formation temperature of PCDT/TAs under the pyrolysis or combustion conditions. Due to the absence of the available experimental rate constants, it is difficult to make a direct comparison of the calculated CVT/SCT rate constants with the experimental values for the reactions of CTPs with H and OH. Our previous studies have shown that the CVT/SCT rate constants of phenol + H → phenoxy + H_2_ and phenol + OH → phenoxy + H_2_O are in good agreement with the available experimental values [34,35]. To be used more effectively, the CVT/SCT rate constants are fitted, and Arrhenius formulas are given in Table 3 for the triophenoxyl-hydrogen abstraction from CTPs by H and in Table 4 for the thiophenoxyl-hydrogen abstraction from CTPs by OH. The pre-exponential factor, the activation energy, and the rate constants can be obtained.

**Table 3:** Arrhenius formulas (in cm^3^·molecule^−1^·s^−1^) for the thiophenoxyl-hydrogen abstraction from chlorothiophenols and thiophenol by H over the temperature range of 600–1200 K.

Reactions	Arrhenius Formulas
Thiophenol + H → C_6_H_5_O + H_2_	*k*(*T*) = (2.43 × 10^−1^) exp (−1877/*T*)
2-CTP + H → 2-CTPR + H_2_	*k*(*T*) = (9.86 × 10^−12^) exp (−2069/*T*)
3-CTP + H → 3-CTPR + H_2_	*k*(*T*) = (1.45 × 10^−11^) exp (−1903/*T*)
4-CTP + H → 4-CTPR + H_2_	*k*(*T*) = (2.23 × 10^−11^) exp (−1753/*T*)
2,3-DCTP + H → 2,3-DCTPR + H_2_	*k*(*T*) = (2.43 × 10^−11^) exp (−2189/*T*)
2,4-DCTP + H → 2,4-DCTPR + H_2_	*k*(*T*) = (2.84 × 10^−11^) exp (−2229/*T*)
2,5-DCTP + H → 2,5-DCTPR + H_2_	*k*(*T*) = (1.12 × 10^−11^) exp (−2105/*T*)
2,6-DCTP + H → 2,6-DCTPR + H_2_	*k*(*T*) = (4.12 × 10^−11^) exp (−2707/*T*)
3,4-DCTP + H → 3,4-DCTPR + H_2_	*k*(*T*) = (4.02 × 10^−11^) exp (−1513/*T*)
3,5-DCTP + H → 3,5-DCTPR + H_2_	*k*(*T*) = (3.42 × 10^−11^) exp (−1566/*T*)
2,3,4-TCTP + H → 2,3,4-TCTPR + H_2_	*k*(*T*) = (3.42 × 10^−11^) exp (−1566/*T*)
2,3,5-TCTP + H → 2,3,5-TCTPR + H_2_	*k*(*T*) = (1.67 × 10^−11^) exp (−1866/*T*)
2,3,6-TCTP + H → 2,3,6-TCTPR + H_2_	*k*(*T*) = (1.47 × 10^−11^) exp (−2365/*T*)
2,4,5-TCTP + H → 2,4,5-TCTPR + H_2_	*k*(*T*) = (2.24 × 10^−11^) exp (−2689/*T*)
2,4,6-TCTP + H → 2,4,6-TCTPR + H_2_	*k*(*T*) = (1.43 × 10^−11^) exp (−2155/*T*)
3,4,5-TCTP + H → 3,4,5-TCTPR + H_2_	*k*(*T*) = (2.03 × 10^−11^) exp (−2476/*T*)
2,3,4,5-TeCTP + H → 2,3,4,5-TeCTPR + H_2_	*k*(*T*) = (2.41 × 10^−11^) exp (−1817/*T*)
2,3,4,6-TeCTP + H → 2,3,4,6-TeCTPR + H_2_	*k*(*T*) = (1.72 × 10^−11^) exp (−2710/*T*)
2,3,5,6-TeCTP + H → 2,3,5,6-TeCTPR + H_2_	*k*(*T*) = (4.70 × 10^−11^) exp (−2893/*T*)
PCTP + H → PCTPR + H_2_	*k*(*T*) = (3.07 × 10^−11^) exp (−2865/*T*)

The chlorine substitution pattern of thiophenol strongly affects the CVT/SCT rate constants. At a given temperature, the calculated CVT/SCT rate constants for the thiophenoxyl-hydrogen abstraction from 2-CTP by H or OH radical is smaller than those of the thiophenoxyl-hydrogen abstraction from 3-CTP and 4-CTP by H or OH radical, respectively. The calculated CVT/SCT rate constants for the thiophenoxyl-hydrogen abstraction from 2,3-DCTP, 2,4-DCTP, 2,5-DCTP and 2,6-DCTP by H or OH radical are smaller than those of the thiophenoxyl-hydrogen abstraction from 3,4-DCTP and 3,5-DCTP by H or OH radical, respectively. The CVT/SCT rate constants for the thiophenoxyl-hydrogen abstraction from 2,3,4-TCTP, 2,3,5-TCTP, 2,3,6-TCTP, 2,4,5-TCTP and 2,4,6-TCTP by H or OH are smaller than that of the thiophenoxyl-hydrogen abstraction from 3,4,5-TCTP by H or OH, respectively. For example, at 1000 K, the CVT/SCT rate constants are 2.54 × 10^−12^, 1.35 × 10^−^^12^, 1.49 × 10^−^^12^, 1.63 × 10^−^^12^, 1.68 × 10^−^^12^ cm^3^·molecule^−1^·s^−1^ for reactions of 2,3,4-TCP, 2,3,5-TCP, 2,3,6-TCP, 2,4,5-TCP and 2,4,6-TCP with H, while the value is 3.82 × 10^−^^12^ cm^3^·molecule^−1^·s^−1^ for that from 3,4,5-TCP with H. Similarly, at 1000 K, the CVT/SCT rate constants are 2.99 × 10^−^^15^, 1.38 × 10^−^^15^, 2.76 × 10^−^^15^, 2.44 × 10^−^^15^, 1.20 × 10^−^^16^ cm^3^·molecule^−1^·s^−^^1^ for reactions of 2,3,4-TCP, 2,3,5-TCP, 2,3,6-TCP, 2,4,5-TCP and 2,4,6-TCP with OH, while the value is 3.44 × 10^−^^14^ cm^3^·molecule^−1^·s^−1^ for that from 3,4,5-TCP with OH. This perfectly matches the structural and thermodynamic analysis above that the chlorine substitution at the *ortho-*position of CTPs increases the strength of the S–H bonds and decreases its reactivity.

**Table 4:** Arrhenius formulas (in cm^3^·molecule^−1^·s^−1^) for the triophenoxyl-hydrogen abstraction from chlorothiophenols and thiophenol by OH over the temperature range of 600–1200 K.

Reactions	Arrhenius Formulas
Thiophenol + OH → C_6_H_5_O + H_2_O	*k*(*T*) = (1.25 × 10^−11^) exp (−5356/*T*)
2-CTP + OH → 2-CTPR + H_2_O	*k*(*T*) = (2.00 × 10^−13^) exp (−6304/*T*)
3-CTP + OH → 3-CTPR + H_2_O	*k*(*T*) = (3.66 × 10^−12^) exp (−5632/*T*)
4-CTP + OH → 4-CTPR + H_2_O	*k*(*T*) = (1.40 × 10^−11^) exp (−5025/*T*)
2,3-DCTP + OH → 2,3-DCTPR + H_2_O	*k*(*T*) = (1.13 × 10^−12^) exp (−5934/*T*)
2,4-DCTP + OH → 2,4-DCTPR + H_2_O	*k*(*T*) = (6.96 × 10^−14^) exp (−5861/*T*)
2,5-DCTP + OH → 2,5-DCTPR + H_2_O	*k*(*T*) = (9.41 × 10^­13^) exp (−6074/*T*)
2,6-DCTP + OH → 2,6-DCTPR + H_2_O	*k*(*T*) = (9.90 × 10^−14^) exp (−6661/*T*)
3,4-DCTP + OH → 3,4-DCTPR + H_2_O	*k*(*T*) = (7.20 × 10^−12^) exp (−5460/*T*)
3,5-DCTP + OH → 3,5-DCTPR + H_2_O	*k*(*T*) = (1.99 × 10^−12^) exp (−5706/*T*)
2,3,4-TCTP + OH → 2,3,4-TCTPR + H_2_O	*k*(*T*) = (1.32 × 10^−12^) exp (−6059 /*T*)
2,3,5-TCTP + OH → 2,3,5-TCTPR + H_2_O	*k*(*T*) = (1.32 × 10^−12^) exp (−6839/*T*)
2,3,6-TCTP + OH → 2,3,6-TCTPR + H_2_O	*k*(*T*) = (3.89 × 10^−12^) exp (−7354/*T*)
2,4,5-TCTP + OH → 2,4,5-TCTPR + H_2_O	*k*(*T*) = (1.17 × 10^−12^) exp (−6143/*T*)
2,4,6-TCTP + OH → 2,4,6-TCTPR + H_2_O	*k*(*T*) = (1.47 × 10^−13^) exp (−7076/*T*)
3,4,5-TCTP + OH → 3,4,5-TCTPR + H_2_O	*k*(*T*) = (9.80 × 10^−12^) exp (−5620/*T*)
2,3,4,5-TeCTP + OH → 2,3,4,5-TeCTPR + H_2_O	*k*(*T*) = (1.86 × 10^−13^) exp (−6366/*T*)
2,3,4,6-TeCTP + OH → 2,3,4,6-TeCTPR + H_2_O	*k*(*T*) = (6.18 × 10^-13^) exp (−7325/*T*)
2,3,5,6-TeCTP + OH → 2,3,5,6-TeCTPR + H_2_O	*k*(*T*) = (2.33 × 10^−13^) exp (−7239/*T*)
PCTP + OH → PCTPR + H_2_O	*k*(*T*) = (1.09 × 10^−13^) exp (−7015/*T*)

For a given thiochlorophenol, the CVT/SCT rate constants for the thiophenoxyl-hydrogen abstraction by H are noticeably larger than those of the thiophenoxyl-hydrogen abstraction by OH over the whole studied temperature range. For example, at 1000 K, the CVT/SCT rate constant of the thiophenoxyl-hydrogen abstraction from 2,3-DCTP by H is 2.66 × 10^−^^12^ cm^3^·molecule·s^−1^, whereas the value is 2.91 × 10^−^^15^ cm^3^·molecule·s^−1^ for the thiophenoxyl-hydrogen abstraction from 2,3-DCTP by OH. This is consistent with thermodynamic analysis: Thiophenoxyl-hydrogen abstraction from CTPs by H is more efficient than the thiophenoxyl-hydrogen abstraction by OH.

Comparison with the previous studies of phenoxyl-hydrogen abstraction by H and OH from CPs shows that the CVT/SCT rate constant for the reaction of CTP with H is consistently larger than that of corresponding CP with H at a given temperature [34], whereas the CVT/SCT rate constant for the reaction of CTP with OH is consistently smaller than that of corresponding CP with OH [35]. This reconfirms thermodynamic analysis that the thiophenoxyl-hydrogen abstraction from CTPs by H is more efficient than the thiophenoxyl-hydrogen abstraction by H and the thiophenoxyl-hydrogen abstraction by OH is less efficient than the phenoxyl-hydrogen abstraction by OH.

## 3. Experimental Section 

### 3.1. Density Functional Theory

The Gaussian 09 program [38] was used to perform all the calculations on the geometries, energies, frequencies for stationary points (reactants, prereactive intermediates, transition states, and products). The MPWB1K method is a hybrid density functional theory (HDFT) model with excellent performance in thermochemistry, thermochemical kinetics, hydrogen bonding and weak interactions [39]. This method has been successfully performed for formation of CPRs from the complete series reactions of 19 CP congeners with H and OH radicals [34,35]. As a serious ongoing work, it is important to use a consistent method for the species involved in the formation of CTPRs from CTPs with H and OH radicals and compare the formation potential of CTPRs and CTPs. Geometry optimizations were optimized at the MPWB1K/6-31+G(d,p) level. The vibrational frequencies were also calculated at the same level to determine the nature of the stationary points, the zero-point energy (ZPE), and the thermal contributions to the free energy of activation. The intrinsic reaction coordinate (IRC) calculations were further carried out at the MPWB1K/6-31+G(d,p) level to confirm that the transition state connects to the right minima along the reaction path [40]. For a more accurate evaluation of the energy parameters, a more flexible basis set, 6-311+G(3df,2p), was employed to determine the single-point energies of the various species. The profiles of the potential energy surface were constructed at the MPWB1K/6-311+G(3df,2p)//MPWB1K/6-31+G(d,p)level, including ZPE correction. 

### 3.2. Kinetic Calculation

Rate constants in this study over a wide temperature range (600–200 K) were calculated using the canonical variational transition state theory (CVT) with small-curvature tunneling (SCT) correction [41,42,43,44]. To calculate the rate constants, 40 non-stationary points near the transition state along the minimum energy path, 20 points on the reactants side and 20 points on the product side were selected. Rate constant calculations were carried out using the Polyrate 9.7 program [45].

### 3.3. Accuracy Verification

The optimized geometries of thiophenol and the calculated vibrational frequencies of thiophenol and 4-chlorotriophenol at the MPWB1K/6-31+G(d,p) level are consistent with the available experimental values, and the relative deviation remains within 1.0% for the geometry parameters and 9.0% for the vibrational frequencies [37,46,47]. To verify the reliability of the energy parameters, we calculated S–H bond dissociation energy for the reaction of thiophenol → thiophenoxy + H at the MPWB1K/6-311+G(3df,2p)//MPWB1K/6-31+G(d,p) level. The calculated value of 86.51 kcal/mol at 298.15 K and 1.0 atm is in excellent agreement with the corresponding experimental value of 86.5 kcal/mol [48]. From these results, we inferred that accuracy can be expected for the species involved in this study.

## 4. Conclusions

In this study, we investigated the theoretical formation of chlorothiophenoxy radicals (CTPRs) from the complete series reactions of 19 chlorothiophenol (CTP) congeners with H and OH radicals using DFT electronic structure theory and canonical variational transition-state (CVT) theory with the small curvature tunneling (SCT) contribution. Structural parameters were calculated for all the stationary (reactants, prereactive intermediates, transition states, and products). Potential barriers, reaction heats, and rate constants for all the elementary reactions were studied to compare the formation potential of CTPRs from CTPs with H and OH radicals. Comparison of this study with our previous studies of the chlorophenoxy radical (CPR) formation from chlorophenols (CPs) with H and OH radicals were discussed [34,35]. Three specific conclusions can be drawn:

(1) The *ortho* chlorine increases the strength of the S–H bond in CTPs and decreased its reactivity, *i.e*., decreases the formation potential of CTPRs from the *ortho*-substitued CTPs with H and OH radicals.

(2) The triophenoxyl-hydrogen abstraction from CTPs by H is more efficient than the phenoxyl-hydrogen abstraction from CPs by H, whereas the thiophenoxyl-hydrogen abstraction from CTPs by OH is less impactful than the phenoxyl-hydrogen abstraction from CPs by OH.

(3) Different from reactions of CPs with H and OH, the thiophenoxyl-hydrogen abstraction from CTPs by H can occur more readily than the thiophenoxyl-hydrogen abstraction by OH radical.

The obtained results can support the important input parameters for the PCDT/TA control models in the environment, and be used for future estimates of PCDT/TAs emission quantity based on the well estimated PCDT/TA inventory.

## Supplementary Materials

MPWB1K/6-31+G(d,p) optimized structures of CTPs and CTPRs. Electron density from total SCF density of 2-CTP, 2-CP, 3-CTP and 3-CP at MPWB1K/6-311+G(3df,2p) level. NBO charge of S and H atoms of CTPs, NBO charge O and H atoms of CPs and HOMO-LOMO gap of CTPs and CPs at MPWB1K/6-31+G(d,p) level. CVT/SCT rate constants for the thiophenoxyl-hydrogen abstraction from CTPs by H and OH radicals. Supplementary materials can be found at http://www.mdpi.com/1422-0067/16/08/18714/s1.
