# Supplementary material for: Formation of Chlorotriophenoxy Radicals from Complete Series Reactions of Chlorotriophenols with H and OH Radicals
Source: Int J Mol Sci. 2015 Aug 11;16(8):18714–31. doi: 10.3390/ijms160818714 (PMC4581267; doi:10.3390/ijms160818714)
Supplement: Supplementary file 1 [file ijms-16-18714-s001.pdf]

Supplementary Information

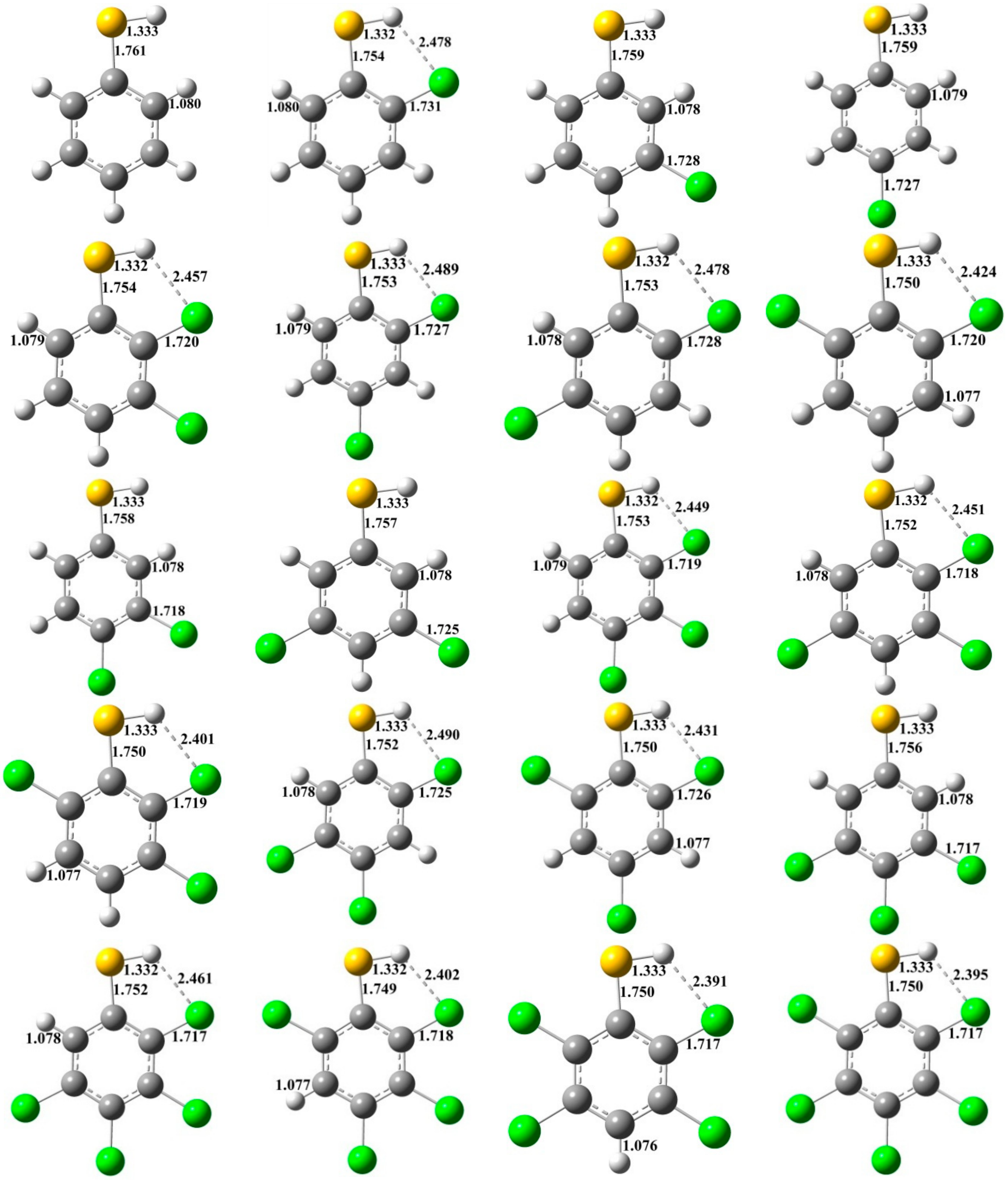

Figure S1. MPWB1K/6-31+G(d,p) optimized geometries for chlorothiophenols and thiophenol. Distances are in angstroms. Gray sphere, C; White sphere, H; Yellow sphere, S; Green sphere, Cl. (For interpretation of the references to colour in this figure legend, the reader is referred to the web version of this article.).

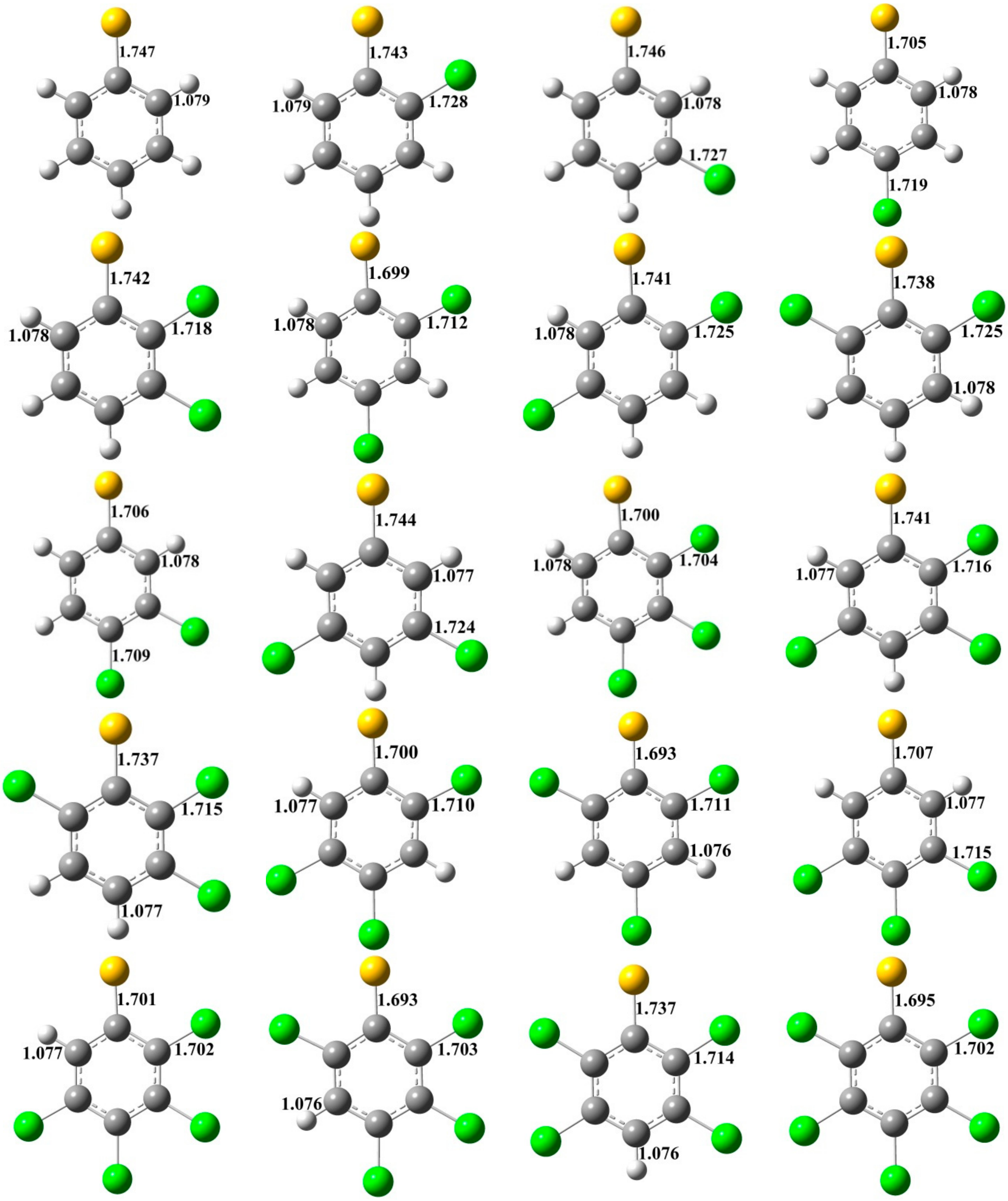

Figure S2. MPWB1K/6-31+G(d,p) optimized geometries for chlorothiophenoxy radicals and thiophenoxy radical. Distances are in angstroms. Gray sphere, C; White sphere, H; Yellow sphere, S; Green sphere, Cl. (For interpretation of the references to colour in this figure legend, the reader is referred to the web version of this article.).

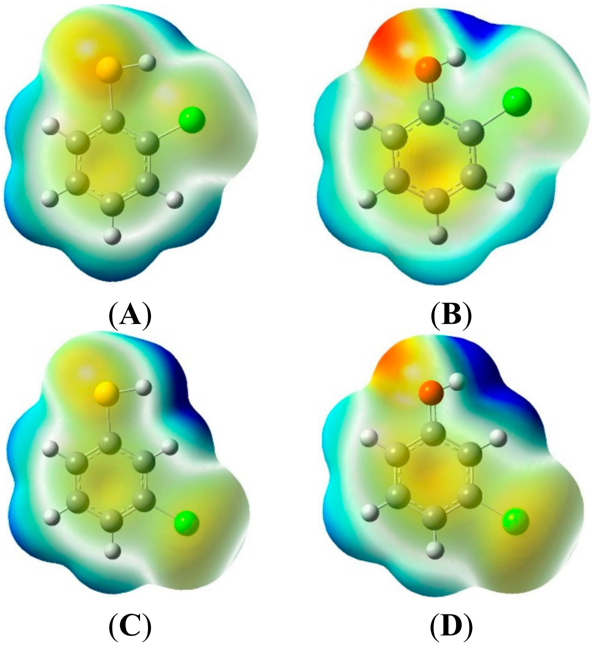

Figure S3. Electron density from total SCF density of (**A**) 2-CTP; (**B**) 2-CP; (**C**) 3-CTP and (**D**) 3-CP at MPWB1K/6-311+G(3df,2p) level (positive region in blue; negative region in red). This is mapped on the surface of molecular electron density at 0.003 e a.u.$^{-3}$.

Table S1. NBO charge (in e) of S and H atoms of CTPs (NBO_S and NBO_H), NBO charge of O and H atoms of CPs (NBO_O and NBO_H) and HOMO-LOMO gap (in a.u.) of CTPs and CPs at MPWB1K/6-31+G(d,p) level. Results of CPs are in brackets.

CTP (CP)	NBO_S (NBO_O)	NBO_H	HOMO-LOMO Gap
Thiophenol (phenol)	0.038 (−0.723)	0.150 (0.513)	0.26891 (0.28138)
2-CTP (2-CP)	0.053 (−0.717)	0.166 (0.523)	0.26548 (0.27823)
3-CTP (3-CP)	0.051 (−0.718)	0.153 (0.516)	0.26606 (0.27809)
4-CTP (4-CP)	0.046 (−0.720)	0.152 (0.515)	0.25943 (0.26886)
2,3-DCTP (2,3-DCP)	0.057 (−0.713)	0.167 (0.524)	0.26417 (0.27791)
2,4-DCTP (2,4-DCP)	0.058 (−0.714)	0.167 (0.524)	0.25649 (0.26616)
2,5-DCTP (2,5-DCP)	0.063 (−0.711)	0.167 (0.525)	0.26263 (0.26331)
2,6-DCTP (2,6-DCP)	0.082 (−0.702)	0.161 (0.526)	0.26366 (0.27502)
3,4-DCTP (3,4-DCP)	0.055 (−0.715)	0.154 (0.518)	0.25704 (0.26664)
3,5-DCTP (3,5-DCP)	0.063 (−0.712)	0.154 (0.519)	0.26579 (0.27917)
2,3,4-TCTP (2,3,4-TCP)	0.066 (−0.711)	0.168 (0.525)	0.25648 (0.26317)
2,3,5-TCTP (2,3,5-TCP)	0.072 (−0.459)	0.168 (0.356)	0.26167 (0.27520)
2,3,6-TCTP (2,3,6-TCP)	0.090 (−0.699)	0.152 (0.527)	0.26155 (0.27019)
2,4,5-TCTP (2,4,5-TCP)	0.068 (−0.710)	0.168 (0.526)	0.25380 (0.26315)
2,4,6-TCTP (2,4,6-TCP)	0.087 (−0.700)	0.162 (0.527)	0.25454 (0.26363)
3,4,5-TCTP (3,4,5-TCP)	0.065 (−0.711)	0.155 (0.520)	0.25634 (0.26592)
2,3,4,5-TeCTP (2,3,4,5-TeCP)	0.074 (−0.707)	0.169 (0.526)	0.25433 (0.26360)
2,3,4,6-TeCTP (2,3,4,6-TeCP)	0.093 (−0.697)	0.163 (0.528)	0.25306 (0.26131)
2,3,5,6-TeCTP (2,3,5,6-TeCP)	0.098 (−0.695)	0.161 (0.528)	0.26088 (0.27181)
PCTP (PCP)	0.098 (−0.694)	0.162 (0.528)	0.25329 (0.26132)

Table S2. CVT/SCT Rate Constants (in $cm^3 \cdot molecule^{-1} \cdot s^{-1}$) for the thiophenoxyl-hydrogen abstraction from chlorothiophenols and thiophenol by H over the temperature range of 600–1200 K.

**T (K)**	**CVT/SCT Rate Constants**			
	Thiophenol + H	2-CTP + H	3-CTP + H	4-CTP + H
600	1.12×10^{-12}	3.31×10^{-13}	6.42×10^{-13}	1.27×10^{-12}
700	1.63×10^{-12}	5.05×10^{-13}	9.33×10^{-13}	1.78×10^{-12}
800	2.22×10^{-12}	7.07×10^{-13}	1.28×10^{-12}	2.37×10^{-12}
900	2.89×10^{-12}	9.44×10^{-13}	1.67×10^{-12}	3.04×10^{-12}
1000	3.64×10^{-12}	1.22×10^{-12}	2.11×10^{-12}	3.78×10^{-12}
1100	4.47×10^{-12}	1.53×10^{-12}	2.61×10^{-12}	4.60×10^{-12}
1200	5.38×10^{-12}	1.87×10^{-12}	3.15×10^{-12}	5.49×10^{-12}
	2,3-DCTP + H	2,4-DCTP + H	2,5-DCTP + H	2,6-DCTP + H
600	6.77×10^{-13}	7.33×10^{-13}	3.47×10^{-13}	4.62×10^{-13}
700	1.04×10^{-12}	1.15×10^{-12}	5.57×10^{-13}	8.53×10^{-13}
800	1.49×10^{-12}	1.67×10^{-12}	7.66×10^{-13}	1.42×10^{-12}
900	2.03×10^{-12}	2.29×10^{-12}	1.02×10^{-12}	1.92×10^{-12}
1000	2.66×10^{-12}	3.00×10^{-12}	1.32×10^{-12}	2.72×10^{-12}
1100	3.39×10^{-12}	3.81×10^{-12}	1.67×10^{-12}	3.48×10^{-12}
1200	4.22×10^{-12}	4.71×10^{-12}	2.06×10^{-12}	4.54×10^{-12}
	3,4-DCTP + H	3,5-DCTP + H	2,3,4-TCTP + H	2,3,5-TCTP + H
600	3.48×10^{-12}	2.73×10^{-12}	9.24×10^{-13}	3.14×10^{-13}
700	4.49×10^{-12}	3.54×10^{-12}	9.79×10^{-13}	4.74×10^{-13}
800	5.68×10^{-12}	4.52×10^{-12}	1.41×10^{-12}	7.10×10^{-13}
900	7.06×10^{-12}	5.66×10^{-12}	1.93×10^{-12}	1.00×10^{-12}
1000	8.62×10^{-12}	6.96×10^{-12}	2.54×10^{-12}	1.35×10^{-12}
1100	1.04×10^{-11}	8.44×10^{-12}	3.24×10^{-12}	1.76×10^{-12}
1200	1.23×10^{-11}	1.01×10^{-11}	4.03×10^{-12}	2.22×10^{-12}
	2,3,6-TCTP + H	2,4,5-TCTP + H	2,4,6-TCTP + H	3,4,5-TCTP + H
600	2.72×10^{-13}	4.17×10^{-13}	3.93×10^{-13}	1.26×10^{-12}
700	4.67×10^{-13}	6.45×10^{-13}	5.13×10^{-13}	1.75×10^{-12}
800	7.34×10^{-13}	9.24×10^{-13}	8.15×10^{-13}	2.31×10^{-12}
900	1.08×10^{-12}	1.25×10^{-12}	1.20×10^{-12}	3.01×10^{-12}
1000	1.49×10^{-12}	1.63×10^{-12}	1.68×10^{-12}	3.82×10^{-12}
1100	1.99×10^{-12}	2.05×10^{-12}	2.24×10^{-12}	4.73×10^{-12}
1200	2.55×10^{-12}	2.52×10^{-12}	2.90×10^{-12}	5.75×10^{-12}
	2,3,4,5-TeCTP + H	2,3,4,6-TeCTP + H	2,3,5,6-TeCTP + H	PCTP + H
600	6.33×10^{-13}	1.98×10^{-13}	3.98×10^{-13}	2.71×10^{-13}
700	9.36×10^{-13}	3.51×10^{-13}	7.37×10^{-13}	5.10×10^{-13}
800	1.34×10^{-12}	5.55×10^{-13}	1.21×10^{-12}	8.29×10^{-13}
900	1.81×10^{-12}	8.13×10^{-13}	1.83×10^{-12}	1.21×10^{-12}
1000	2.34×10^{-12}	1.12×10^{-12}	2.57×10^{-12}	1.68×10^{-12}
1100	2.93×10^{-12}	1.49×10^{-12}	3.45×10^{-12}	2.28×10^{-12}
1200	3.58×10^{-12}	1.90×10^{-12}	4.41×10^{-12}	3.06×10^{-12}

Table S3. CVT/SCT rate constants (in $cm^3 \cdot molecule^{-1} \cdot s^{-1}$) for the thiophenoxyl-hydrogen abstraction from chlorothiophenols and thiophenol by OH over the temperature range of 600–1200 K.

T (K)	CVT/SCT Rate Constants			
	Thiophenol + OH	2-CTP + OH	3-CTP + OH	4-CTP + OH
600	1.81×10^{-15}	5.98×10^{-18}	3.36×10^{-16}	3.52×10^{-15}
700	5.72×10^{-15}	2.38×10^{-17}	1.13×10^{-15}	1.03×10^{-14}
800	1.43×10^{-14}	7.05×10^{-17}	2.96×10^{-15}	2.43×10^{-14}
900	3.02×10^{-14}	1.70×10^{-16}	6.53×10^{-15}	4.91×10^{-14}
1000	5.70×10^{-14}	3.56×10^{-16}	1.27×10^{-14}	8.89×10^{-14}
1100	9.83×10^{-14}	6.67×10^{-16}	2.24×10^{-14}	1.49×10^{-13}
1200	1.58×10^{-13}	1.15×10^{-15}	3.69×10^{-14}	2.33×10^{-13}
	2,3-DCTP + OH	2,4-DCTP + OH	2,5-DCTP + OH	2,6-DCTP + OH
600	6.26×10^{-17}	4.34×10^{-18}	4.06×10^{-17}	1.63×10^{-18}
700	2.28×10^{-16}	1.56×10^{-17}	1.56×10^{-16}	7.05×10^{-18}
800	6.32×10^{-16}	4.26×10^{-17}	4.46×10^{-16}	2.22×10^{-17}
900	1.45×10^{-15}	9.68×10^{-17}	1.04×10^{-15}	5.64×10^{-17}
1000	2.91×10^{-15}	1.93×10^{-16}	2.11×10^{-15}	1.23×10^{-16}
1100	5.27×10^{-15}	3.46×10^{-16}	3.84×10^{-15}	2.38×10^{-16}
1200	8.85×10^{-15}	5.77×10^{-16}	6.45×10^{-15}	4.22×10^{-16}
	3,4-DCTP + OH	3,5-DCTP + OH	2,3,4-TCTP + OH	2,3,5-TCTP + OH
600	8.79×10^{-16}	1.61×10^{-16}	5.91×10^{-17}	1.59×10^{-17}
700	2.85×10^{-15}	5.54×10^{-16}	2.22×10^{-16}	7.32×10^{-17}
800	7.25×10^{-15}	1.47×10^{-15}	6.28×10^{-16}	2.40×10^{-16}
900	1.56×10^{-14}	3.27×10^{-15}	1.47×10^{-15}	6.24×10^{-16}
1000	2.97×10^{-14}	6.41×10^{-15}	2.99×10^{-15}	1.38×10^{-15}
1100	5.16×10^{-14}	1.14×10^{-14}	5.47×10^{-15}	2.68×10^{-15}
1200	8.36×10^{-14}	1.88×10^{-14}	9.27×10^{-15}	4.77×10^{-15}
	2,3,6-TCTP + OH	2,4,5-TCTP + OH	2,4,6-TCTP + OH	3,4,5-TCTP + OH
600	2.25×10^{-17}	4.58×10^{-17}	1.22×10^{-18}	9.19×10^{-16}
700	1.01×10^{-16}	1.75×10^{-16}	5.77×10^{-18}	3.09×10^{-15}
800	3.26×10^{-16}	5.01×10^{-16}	1.96×10^{-17}	8.06×10^{-15}
900	8.40×10^{-16}	1.19×10^{-15}	5.27×10^{-17}	1.77×10^{-14}
1000	2.76×10^{-15}	2.44×10^{-15}	1.20×10^{-16}	3.44×10^{-14}
1100	5.37×10^{-15}	4.51×10^{-15}	2.43×10^{-16}	6.09×10^{-14}
1200	9.56×10^{-15}	7.70×10^{-15}	4.45×10^{-16}	1.00×10^{-13}
	2,3,4,5-TeCTP + OH	2,3,4,6-TeCTP + OH	2,3,5,6-TeCTP + OH	PCTP + OH
600	5.04×10^{-18}	3.32×10^{-18}	1.47×10^{-18}	9.96×10^{-19}
700	2.02×10^{-17}	1.71×10^{-17}	7.27×10^{-18}	4.71×10^{-18}
800	6.01×10^{-17}	6.13×10^{-17}	2.54×10^{-17}	1.58×10^{-17}
900	1.47×10^{-16}	1.71×10^{-16}	6.98×10^{-17}	4.22×10^{-17}
1000	3.10×10^{-16}	3.97×10^{-16}	1.62×10^{-16}	9.53×10^{-17}
1100	5.87×10^{-16}	8.10×10^{-16}	3.32×10^{-16}	1.91×10^{-16}
1200	1.02×10^{-15}	1.49×10^{-15}	6.17×10^{-16}	3.46×10^{-16}
